# Gram-stained smear in the diagnosis of acute urethritis: is it coming to an end?

**DOI:** 10.1590/1806-9282.20231532

**Published:** 2024-04-22

**Authors:** Mehmet Sarier, Esin Kasap

**Affiliations:** 1 Istinye University, Department of Urology – İstanbul, Turkey.; 2 Medical Park Hospital, Department of Urology – Antalya, Turkey.; 3 University of Health Science, Tepecik Education and Research Hospital, Department of Obstetrics and Gynecology – İzmir, Turkey.

Acute urethritis is the most common infection of the male genital tract. Approximately 89 million new cases of non-gonococcal urethritis (NGU) and 62 million new cases of gonococcal urethritis (GU) are reported globally every year, and these numbers continue to increase^1^. Acute urethritis is most commonly caused by sexually transmitted pathogens. The three cardinal symptoms are urethral discharge, dysuria, and itching. The traditional diagnostic method for acute urethritis is a Gram-stained smear (GSS) of urethral discharge. GSS is widely used because it is of low cost and is easy to perform. Not only does GSS diagnose acute urethritis but it also allows the dichotomization of cases as GU caused by *Neisseria gonorrhoeae* with the detection of gram-negative diplococci or NGU in their absence^2^. However, GSS is a test susceptible to inter- and intra-observer errors.

In the classical approach, the treatment of acute urethritis is managed through GSS. GSS inevitably leads the clinician to empirical treatment, especially in cases of NGU, as the specific identification of NGU pathogens by conventional methods is a long process. However, treatment failure occurs in up to 20% of NGU patients who receive empirical treatment based on the results of GSS^3^. Moreover, empirical treatment practices also contribute to the development of resistant strains. Antibiotic resistance in *N. gonorrhoeae* and *Mycoplasma genitalium* is a serious public health problem^4^, and *M. genitalium* alone is responsible for 41% of recurrent urethritis cases^5^.

The widespread use of nucleic acid amplification tests such as polymerase chain reaction (PCR) has brought about significant advances in the management of acute urethritis^6^. PCR enables the rapid identification of multiple pathogens from a single sample with high sensitivity and specificity and has become the gold standard for identifying urethritis pathogens^7^. PCR also allowed inquiry into the effectiveness of GSS. The inability of GSS to detect coinfections of NGU pathogens with GU is an important limitation. GSS is also ineffective in urethritis patients with low inflammation, some of whom may even be asymptomatic. In a recently published study, 68.7% of urethritis cases evaluated by PCR did not have apparent urethral discharge, making it difficult to detect these cases with GSS^8^. This results in misdiagnosis and patients continuing to act as a vector of the contagion.

Traditionally, the threshold for GSS is ≥5 polymorphonuclear leucocytes (PMNL)/high-power field (HPF)^9^. However, a PCR confirmation study demonstrated that GSS had 55.6% sensitivity in the diagnosis of NGU when using a threshold of ≥5 PMNL/HPF in cases of acute urethritis^10^. Current guidelines recommend a GSS threshold of ≥2 PMNL/HPF for the diagnosis of NGU^11^. Considering that the frequency of NGU pathogens in acute urethritis is above 80%^12^, GSS may be seriously inadequate in identifying the majority of acute urethritis cases.

In terms of cost, GSS and PCR are not comparable at present. However, the risks of misdiagnosis, antibiotic resistance, and recurrent urethritis associated with GSS warrant a critical scrutiny of its cost-effectiveness. In our view, GSS has fulfilled its role in the diagnosis of acute urethritis. Going forward, PCR assay should be regarded as the first choice for both the diagnosis of acute urethritis and the identification of causative pathogens. In our view, this is, first and foremost, the right approach for public health.
